# *In-Vivo* Effect of Andrographolide on Alveolar Bone Resorption Induced by *Porphyromonas gingivalis* and Its Relation with Antioxidant Enzymes

**DOI:** 10.1155/2013/276329

**Published:** 2013-09-17

**Authors:** Rami Al Batran, Fouad H. Al-Bayaty, Mazen M. Jamil Al-Obaidi

**Affiliations:** Center of Studies for Periodontology, Faculty of Dentistry, Universiti Teknologi MARA (UiTM), 40450 Shah Alam, Selangor Darul Ehsan, Malaysia

## Abstract

Alveolar bone resorption is one of the most important facts in denture construction. *Porphyromonas gingivalis* (*Pg*) causes alveolar bone resorption, and morphologic measurements are the most frequent methods to identify bone resorption in periodontal studies. This study has aimed at evaluating the effect of Andrographolide (AND) on alveolar bone resorption in rats induced by *Pg*. 24 healthy male *Sprague Dawley* rats were divided into four groups as follows: normal control group and three experimental groups challenged orally with *Pg* ATCC 33277 five times a week supplemented with 20 mg/kg and 10 mg/kg of AND for twelve weeks. Alveolar bones of the left and right sides of the mandible were assessed by a morphometric method. The bone level, that is, the distance from the alveolar bone crest to cementumenamel junction (CEJ), was measured using 6.1 : 1 zoom stereomicroscope and software. AND reduced the effect of *Pg* on alveolar bone resorption and decreased the serum levels of Hexanoyl-Lysine (HEL); furthermore the reduced glutathione/oxidised glutathione (GSH/GSSG) ratio in AND treated groups (10 and 20 mg/kg) significantly increased when compared with the *Pg* group (*P* < 0.05). We can conclude that AND suppresses alveolar bone resorption caused by *Pg* in rats.

## 1. Introduction

Periodontal diseases are chronic inflammatory diseases that result in both loss of attachment between teeth and periodontium and osteoclastic resorption of alveolar bone [1]. They are very widespread in humans and are associated with the presence of several species of subgingival microorganisms, particularly Gram-negative anaerobic bacteria. Infection with *Pg* results in a variety of host immune responses [2]. It is not known whether the disease state results when host response is insufficiently protective or, alternatively, if the immune response itself is actively destructive [3]. Both may be true at different times, or different aspects of the immune response may be either protective or destructive [4]. *Pg* is a Gram-negative, anaerobic, and black-pigmented bacterium closely associated with adult periodontitis in humans [5]. It stimulates bone resorption in rat calvaria cultures via its lipopolysaccharide [6] and causes osteoclastic bone resorption in the hind paws of mice and alveolar bone resorption in rats [7].

Oxidative stress causes profound alterations of various biological structures, including tissue damage, lipids, proteins and nucleic acids, and it is involved in numerous infections. Studies have shown that periodontitis induces excessive reactive oxygen species (ROS) production in periodontal tissue [8]. When periodontitis develops, ROS produced in the periodontal lesion diffuse into the blood stream, resulting in the oxidation of blood molecules (circulating oxidative stress) [9]. Recent studies indicate that oxidative mechanisms, including lipid peroxidation, are involved in periodontitis and lipid peroxidation may play an important role in the pathogenesis of periodontitis. The Hexanoyl-Lysine (HEL) is a useful biomarker for detecting and quantifying the earlier stages of lipid peroxidation [10]. Reduced glutathione (GSH) is considered to be one of the most important scavengers of reactive oxygen species (ROS), and its ratio with oxidized glutathione (GSSG) may be used as a marker of oxidative stress [11]. 

*Andrographis paniculata* (Burm. f.) Nees (Acanthaceae) is a long-established therapeutic plant familiar in Southeast Asia and originated from India to Indo-China. The plant is generally recognized as king of bitter. *A. paniculata* contains Andrographolide as the main dynamic code and other codes like 14-deoxy-11, 12-didehydroandrographolide, and 14-deoxyandrographolideetc. The plant is also known for its hypotensive [12], antihyperglycemic [13], phagocytic [14], wound healing [15], gastroprotective [16] and hyperlipidemia properties [17]. In the present work, we hypothesized alveolar bone resorption with alteration of oxidative stress in serum caused by oral challenge with *Pg*. The main aim of this study was to assess the effect of AND intake on alveolar bone resorption, beside determination of HEL and GSH : GSSG ratio in the blood serum of rats in order to use this ratio as a potential marker of oxidative stress in rats fed a *Pg*.

## 2. Materials and Methods

### 2.1. Chemicals

Andrographolide was purchased from (Sigma Aldrich, USA) and *Porphyromonas gingivalis *from (ATCC, USA).

### 2.2. Culturing Bacteria

Under anaerobic condition, *Pg* ATCC strain 33277 was cultured on anaerobic blood agar plates (Becton Dickinson Co) in an aerobic chamber (Coy Laboratory Products Inc.) with 85% N_2_, 5% H_2_, and 10% CO_2_ from 3 to 5 days and then inoculated into Schaedler Broth (Difco Laboratories) containing hemin and menadione for 24 hours according to the previous protocol [18], and then bacteria were harvested from Schaedler Broth resuspended in phosphate-buffered saline (PBS) and carboxymethylcellulose (CMC) in an anaerobic state for feeding the rats.

### 2.3. Animals

Twenty-four healthy male *Sprague Dawley* rats (6–8 weeks old) weighing between 180 and 200 g were obtained from the animal house in the University of Malaya (Ethic number 28/05/2012. 600-FF PT. 5/2, University Technology Mara). All rats were kept in wire bottomed cages at 25 ± 2°C, given tap water and standard pellet diet, and exposed to a 12 h:12 h light-dark cycle at 50–60% humidity in an animal room. Throughout the experiments, all animals received human care according to the criteria outlined in the “Guide for the Care and Use of Laboratory Animals” prepared by the National Academy of Sciences and published by the National Institute of Health. 

### 2.4. Experimental Design

*Pg* is an anaerobic bacteria most strongly associated with periodontal disease; administration of *Pg* to experimental animals is widely used as a model of periodontal infection.

Rats of Group 1 (normal control group) were orally administrated with sterile distilled water. 

Groups 2–4 were challenged orally using gavage with *Pg* (0.2 mL of 1.5 × 10^×12^ bacterial cells/mL in 2% (CMC) with (PBS)) five times a week over 12 weeks to induce alveolar bone resorption. Constant exposure of this concentration of *Pg* induces periodontal changes in the alveolar bone [19].

Rats of Group 2 (negative control group) were orally challenged with *Pg* five times a week. 

Rats of Group 3 (AND-treated group) as low dose were orally administrated with AND at a dose of 10 mg/kg daily for 12 weeks. AND was properly dissolved in sterile distilled water.

Rats of Groups 4 (AND-treated group) as high dose were orally administrated with AND at a dose of 20 mg/kg daily for 12 weeks. AND was properly dissolved in sterile distilled water.

### 2.5. Blood Sampling

At the end of the experimental period, the animals were euthanized with Ketamine (30 mg/kg, 100 mg/mL) and Xylazil (3 mg/kg, 100 mg/mL) anesthesia [20]. Blood samples were collected by cardiac puncture from 24-hour fasted rats. Blood was allowed to clot, and serum samples were separated by centrifugation at 1500 g for 15 minutes at 4°C [21]. Serum samples were stored at −80°C until subsequent analysis. 

### 2.6. Measurement of Glutathione and HEL Levels in Serum

Serum samples from each group were used to evaluate the concentration of HEL with an enzyme-linked immunosorbent assay (ELISA) kit according to the manufacture protocol (Northwest Life Science Specialties, LLC, Vancouver, USA). The levels of total glutathione (GSH + GSSG levels) and GSSG were quantified by colorimetric assay kits (Promega, USA). Oxidized-form glutathione was determined after blocking GSH with 2-vinylpyridine, and the GSH/GSSG ratio in each sample was calculated [22].

### 2.7. Evaluation of Alveolar Bone Resorption

Alveolar bone resorption of the left and right sides of the mandible was assessed by a morphometric method. Euthanasia of the rats was done at the end of the experiment with an intramuscular injection of 2% xylazine 10 mg/kg body weight and then anesthetized with 5% ketamine hydrochloride. The mandible was separated from the remaining skull using bone cutters. The mandible was defleshed by first placing them in boiling water for 15 minutes and then mechanically removing the superficial flesh, and then exposed overnight to 8% hydrochloric acid. After air drying, the mandible was immersed in methylene blue stain 1% for 1 minute. The area of alveolar bone loss was evaluated morphometrically by measuring the distance between the CEJ to the alveolar bone crest in the three molars on the buccal and lingual parts. All measurements were made along the long axis of the roots [23]. The measurements were performed electronically using 6.1:1 zoom stereomicroscope (Olympus SZ2-LISTD, Tokyo, Japan) and Image-Pro Plus 6.2 (Media Cybernetics Inc., Maryland, USA) software. Before performing alveolar bone loss measurements, the examiner was trained and calibrated. Double measurements of 20 specimens were performed within the period of five days. A very high correlation was obtained between the 2 measurements, and verified by intraclass correlation coefficient (ICC = 0.95).

### 2.8. Statistical Analysis

All values were reported as mean ± SEM. Data were analyzed by one-way ANOVA and Tukey's post hoc test for multiple comparison using SPSS 18 (Statistical Package for the Social Sciences) software for windows (SPSS Malaysia). Significance was defined as *P < 0.05.

## 3. Results and Discussion

*Andrographis paniculata* (Burm. f.) Nees is a medicinal plant; traditionally this plant is used as antidiabetes, anti-inflammatory, hepatoprotective, antispasmodic, and antioxidant agents [24, 25]. *A. paniculata* contains major constituents such as diterpenoids, flavonoids, and polyphenol [26]. AND is a major compound and found in abundance in the plant. AND has been used for many years to treat a variety of diseases including bacterial and viral infections. A previous study by Arifullah et al. [27] showed that AND has antibacterial activities. Earlier studies did not show significant toxicity of AND through clinical and histopathology observations [28].

In the present study, AND was fed to the rats at a concentration of 10 and 20 mg/kg.

Table 1 shows the level of HEL and GSH/GSSG ratio of all experimental groups. In the current study, serum HEL levels in the normal control group (Group 1) were significantly lower than the control group (Group 2). Likewise, serum HEL levels in groups fed orally with AND 10 and 20 mg/kg (Group 4 and 5) were significantly lowered compared to control group (Group 2), and this could be due to the effect of AND in inhibiting the effects of *Pg* on the serum HEL level. The systemic increase of antioxidant activity due to AND, resulting in a decrease in circulating lipid peroxidation is conceivable.

Some reports have demonstrated that osteoclast differentiation is stimulated by oxidative stress [29]. These data indicate that oxidative stress plays a crucial role in osteoclast differentiation. 

In the present study, normal group showed significant increase in the levels of GSH/GSSG ratio in serum as compared to control group. Oral administration of AND with 10 and 20 mg/kg significantly increases the GSH/GSSG ratio in serum, which was more potent at doses of 20 mg/kg. This displays that AND improved the antioxidant status. In other words, the reduction in oxidative stress by supplementation with AND may lead to the suppression of osteoclast differentiation induced by *Pg*, and these findings were in agreement with the previous study showing that vitamin C could decrease the GSH/GSSG ratio in rats [30]. 

**Figure 1:**
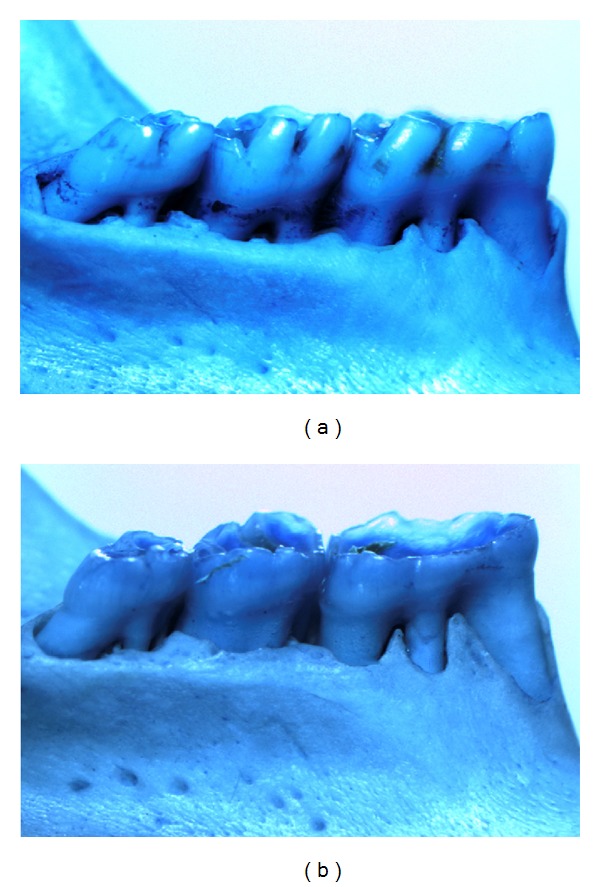
Morphometric analysis of alveolar bone resorption of posterior mandibles. (a) Vehicle and *Pg*, (b) AND 20 mg/kg.

**Figure 2:**
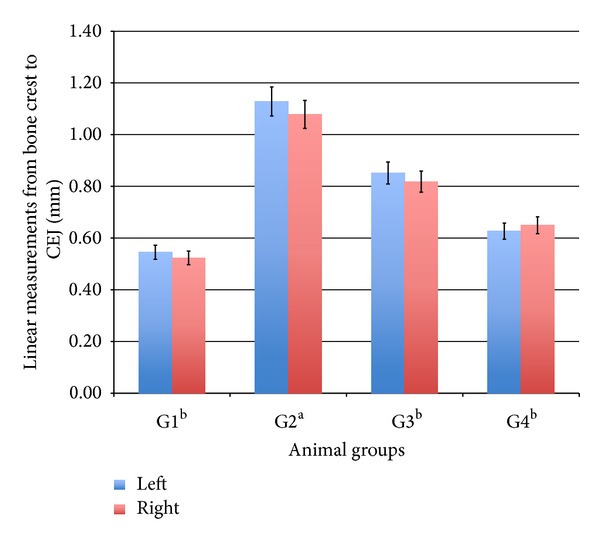
Effect of Andrographolide on alveolar bone resorption of left and right mandibles induced by *Pg*. Blue is left mandible; red is right mandible. Group (1) vehicle; Group (2) vehicle and *Pg*; Group (3) AND 10 mg/kg; Group (4) AND 20 mg/kg. Statistical analysis of the data was carried out using one-way analysis of variance (ANOVA) and Tukey's post hoc test for average comparison on SPSS 18.0. Mean values ± SEM (*n* = 6) were used. Significance was defined as a and b (*P* < 0.05) where a compared to G1 and b compared to G2. Values, which are not struck, do not have significant difference compared to G2.

There is strong evidence that *Pg* may play a significant role in alveolar bone resorption [31]. To determine the degree of local periodontal destruction, we measured the alveolar bone resorption in left and right mandibles by a morphometric method, the distances between the alveolar bone crest to the cementoenamel junction (CEJ), as illustrated in (Figure 1), and our results showed that there was a significant decrease of alveolar bone resorption in the AND (10 and 20 mg/kg) groups than those in the *Pg* group (Figure 2). 

In our study, group challenge orally with *Pg* induced alveolar bone resorption demonstrated by increase in the distance between alveolar bone crest to cementumenamel junction. This indicates that the degree of alveolar bone resorption induced by *Pg* in the groups treated with 20 mg/kg and 10 mg/kg of AND was lower compared to the rats challenged orally with *Pg* alone. Similar findings but with different method to measure the alveolar bone resorption were observed by Sanbe et al. [32]. In addition, the intake of 20 mg/kg and 10 mg/kg AND decreased the distance between alveolar bone crest to cementumenamel junction by 24% and 44% of the left mandible and 24% and 39% of the right mandible, respectively, which demonstrated that AND reduced alveolar bone loss in rats. Further studies with periodontal tissue homogenate needed to understand by which mechanism AND alters the local oxidative marker.

## 4. Conclusions 

In conclusion, our results highlight the effect of AND on alveolar bone resorption caused by *Pg* in a dose-dependent manner. Since the results indicate that a high dose of AND has reduced the amount of alveolar bone loss compared to low dose. *Pg* caused periodontitis that induces excessive production of ROS from inflammatory cells, resulting in circulating oxidative stress. AND treatment was effective in reversing oxidative stress parameters (HEL and GSH/GSSG ratio) to nearly the normal value. The improvement of periodontitis by AND treatment may offer clinical benefits on systemic health by decreasing circulating oxidative stress. Furthermore, elevated oxidative stress is harmful to periodontal health. In these cases, therapeutic approaches to decrease the systemic oxidative stress would be important to improve periodontal health. 

## Figures and Tables

**Table 1:** Effect of Andrographolide on HEL and GSH/GSSG ratio.

Groups	Animal group	HEL (nmol/L)	GSH/GSSG ratio
Group 1	CMC	6.56 ± 0.07^b^	25.24 ± 0.63^b^
Group 2	CMC+ *Pg *	9.13 ± 0.06^a^	17.28 ± 0.14^a^
Group 3	AND 10 mg/kg	7.71 ± 0.07^b^	23.15 ± 0.51^b^
Group 4	AND 20 mg/kg	6.51 ± 0.05^b^	24.34 ± 0.66^b^

Statistical analysis of the data was carried out using one-way analysis of variance (ANOVA) and Tukey's post hoc test for average comparison on SPSS 18.0. Mean values ± SEM (*n* = 6) were used. Significance was defined as a and b (*P* < 0.05) where a compared to G1 and b compared to G2. Values, which are not stuck, do not have significant difference compared to G2.

## References

[1] Grossi SG, Genco RJ, Machtei EE (1995). Assessment of risk for periodontal disease. II. Risk indicators for alveolar bone loss. *Journal of Periodontology*.

[2] Beck J, Garcia R, Heiss G, Vokonas PS, Offenbacher S (1996). Periodontal disease and cardiovascular disease. *Journal of Periodontology*.

[3] Genco RJ (1992). Host responses in periodontal diseases: current concepts. *Journal of Periodontology*.

[4] Shenker BJ (1987). Immunologic dysfunction in the pathogenesis of periodontal diseases. *Journal of Clinical Periodontology*.

[5] Shah HN, Collins MD (1988). Proposal for reclassification of *Bacteroides asaccharolyticus*, *Bacteroides gingivalis*, and *Bacteroides endodontalis* in a new genus, Porphyromonas. *International Journal of Systematic Bacteriology*.

[6] Lamont RJ, Chan A, Belton CM, Izutsu KT, Vasel D, Weinberg A (1995). *Porphyromonas gingival*is invasion of gingival epithelial cells. *Infection and Immunity*.

[7] Evans RT, Klausen B, Ramamurthy NS, Golub LM, Sfintescu C, Genco RJ (1992). Periodontopathic potential of two strains of *Porphyromonas gingival* is in gnotobiotic rats. *Archives of Oral Biology*.

[8] Wei P, Ho K, Ho Y, Wu Y, Yang Y, Tsai C (2004). The investigation of glutathione peroxidase, lactoferrin, myeloperoxidase and interleukin-1*β* in gingival crevicular fluid: Implications for oxidative stress in human periodontal diseases. *Journal of Periodontal Research*.

[9] Waddington RJ, Moseley R, Embery G (2000). Reactive oxygen species: a potential role in the pathogenesis of periodontal diseases. *Oral Diseases*.

[10] Battino M, Bullon P, Wilson M, Newman H (1999). Oxidative injury and inflammatory periodontal diseases: the challenge of anti-oxidants to free radicals and reactive oxygen species. *Critical Reviews in Oral Biology and Medicine*.

[11] Borges I, Machado Moreira EA, Filho DW, De Oliveira TB, Da Silva MBS, Fröde TS (2007). Proinflammatory and oxidative stress markers in patients with periodontal disease. *Mediators of Inflammation*.

[12] Zhang CY, Tan B (1996). Hypotensive activity of aqueous extract of Andrographis paniculata in rats. *Clinical and Experimental Pharmacology and Physiology*.

[13] Zhang X, Tan BK (2000). Antihyperglycaemic and anti-oxidant properties of Andrographis paniculata in normal and diabetic rats. *Clinical and Experimental Pharmacology and Physiology*.

[14] Al- Bayaty FH, Abdulla MA, Abu Hassan MI, Hussein SF (2010). Effects of Malaysian medicinal plants on macrophage functions in vitro study. *Journal of Medicinal Plants Research*.

[15] Al-Bayaty FH, Abdulla MA, Hassan MIA, Ali HM (2012). Effect of Andrographis paniculata leaf extract on wound healing in rats. *Natural Product Research*.

[16] Wasman SQ, Mahmood AA, Chua LS, Alshawsh MA, Hamdan S (2011). Antioxidant and gastroprotective activities of *Andrographis paniculata* (Hempedu Bumi) in Sprague Dawley rats. *Indian Journal of Experimental Biology*.

[17] Nugroho AE, Andrie M, Warditiani NK, Siswanto E, Pramono S, Lukitaningsih E (2012). Antidiabetic and antihiperlipidemic effect of *Andrographis paniculata* (Burm. f.) Nees and andrographolide in high-fructose-fat-fed rats. *Indian Journal of Pharmacology*.

[18] Gibson FC, Yumoto H, Takahashi Y, Chou H-H, Genco CA (2006). Innate immune signaling and *Porphyromonas gingival*is-accelerated atherosclerosis. *Journal of Dental Research*.

[19] Li L (2002). *Porphyromonas gingivalis* infection accelerates the progression of atherosclerosis in a heterozygous apolipoprotein e-deficient murine model. *Circulation*.

[20] Fatemi F, Allameh A, Khalafi H, Ashrafihelan J (2010). Hepatoprotective effects of *γ*-irradiated caraway essential oils in experimental sepsis. *Applied Radiation and Isotopes*.

[21] Hem A, Smith AJ, Solberg P (1998). Saphenous vein puncture for blood sampling of the mouse, rat, hamster, gerbil, guineapig, ferret and mink. *Laboratory Animals*.

[22] Tomofuji T, Ekuni D, Sanbe T (2009). Effects of vitamin C intake on gingival oxidative stress in rat periodontitis. *Free Radical Biology and Medicine*.

[23] Björnsson MJ, Velschow S, Stoltze K, Havemose-Poulsen A, Schou S, Holmstrup P (2003). The influence of diet consistence, drinking water and bedding on periodontal disease in Sprague-Dawley rats. *Journal of Periodontal Research*.

[24] Koteswara Rao Y, Vimalamma G, Venkata Rao C, Tzeng Y (2004). Flavonoids and andrographolides from *Andrographis paniculata*. *Phytochemistry*.

[25] Niranjan A, Tewari SK, Lehri A (2010). Biological activities of Kalmegh (*Andrographis paniculata* Nees) and its active principles—a review. *Indian Journal of Natural Products and Resources*.

[26] Koteswara Rao Y, Vimalamma G, Venkata Rao C, Tzeng Y (2004). Flavonoids and andrographolides from *Andrographis paniculata*. *Phytochemistry*.

[27] Arifullah M (2013). Evaluation of anti-bacterial and anti-oxidant potential of andrographolide and echiodinin isolated from callus culture of *Andrographis paniculata* Nees. *Asian Pacific Journal of Tropical Biomedicine*.

[28] Al Batran R, Al-Bayaty F, Jamil Al-Obaidi MM, Abdulla MA (2013). Acute toxicity and the effect of andrographolide on porphyromonas gingivalis-induced hyperlipidemia in rats. *BioMed Research International*.

[29] Das AS, Mukherjee M, Das D, Mitra C (2009). Protective action of aqueous black tea (*Camellia sinensis*) extract (BTE) against ovariectomy-induced oxidative stress of mononuclear cells and its associated progression of bone loss. *Phytotherapy Research*.

[30] Ekuni D, Tomofuji T, Sanbe T (2009). Vitamin C intake attenuates the degree of experimental atherosclerosis induced by periodontitis in the rat by decreasing oxidative stress. *Archives of Oral Biology*.

[31] Baker PJ, Evans RT, Roopenian DC (1994). Oral infection with *Porphyromonas gingival*is and induced alveolar bone loss in immuncompetent and severe combined immunodeficient mice. *Archives of Oral Biology*.

[32] Sanbe T, Tomofuji T, Ekuni D, Azuma T, Tamaki N, Yamamoto T (2007). Oral administration of vitamin C prevents alveolar bone resorption induced by high dietary cholesterol in rats. *Journal of Periodontology*.

